# High mortality in HIV-infected children diagnosed in hospital underscores need for faster diagnostic turnaround time in prevention of mother-to-child transmission of HIV (PMTCT) programs

**DOI:** 10.1186/s12887-015-0325-8

**Published:** 2015-02-15

**Authors:** Anjuli Wagner, Jennifer Slyker, Agnes Langat, Irene Inwani, Judith Adhiambo, Sarah Benki-Nugent, Ken Tapia, Irene Njuguna, Dalton Wamalwa, Grace John-Stewart

**Affiliations:** Department of Epidemiology, University of Washington, Box 359300, Seattle, WA 98104 USA; Department of Global Health, University of Washington, Box 359931, Seattle, WA 98104 USA; Centers for Disease Control and Prevention (CDC), Mbagathi Road, P.O. Box 54840, Nairobi, 00200 Kenya; Kenyatta National Hospital, Ngong Road, Nairobi, 00202 Kenya; Department of Paediatrics and Child Health, University of Nairobi, P.O. Box 19676, Nairobi, 00202 Kenya; Department of Medicine, University of Washington, Box 359931, Seattle, WA 98104 USA; Departments of Global Health, Medicine, Epidemiology & Pediatrics, University of Washington, Box 359909, Seattle, WA 98104 USA

**Keywords:** Pediatric, Infant, HIV, PMTCT, Hospital, Early infant diagnosis, Delayed diagnosis

## Abstract

**Background:**

Despite expanded programs for prevention of mother-to-child HIV transmission (PMTCT), HIV-infected infants may not be diagnosed until they are ill. Comparing HIV prevalence and outcomes in infants diagnosed in PMTCT programs to those in hospital settings may improve pediatric HIV diagnosis strategies.

**Methods:**

HIV-exposed infants <12 months old were recruited from 9 PMTCT sites in public maternal child health (MCH) clinics or from an inpatient setting in Nairobi, Kenya and tested for HIV using HIV DNA assays. A subset of HIV-infected infants <4.5 months of age was enrolled in a research study and followed for 2 years. HIV prevalence, number needed to test, infant age at testing, and turnaround time for tests were compared between PMTCT programs and hospital sites. Among the enrolled cohort, baseline characteristics, survival, and timing of antiretroviral therapy (ART) initiation were compared between infants diagnosed in PMTCT programs versus hospital.

**Results:**

Among 1,923 HIV-exposed infants, HIV prevalence was higher among infants tested in hospital than PMTCT early infant diagnosis (EID) sites (41% vs. 11%, p < 0.001); the number of HIV-exposed infants needed to test to diagnose one infection was 2.4 in the hospital vs. 9.1 in PMTCT. Receipt of HIV test results was faster among hospitalized infants (7 vs. 25 days, p < 0.001). Infants diagnosed in hospital were older at the time of testing than PMTCT diagnosed infants (5.0 vs. 1.6 months, respectively, p < 0.001).

In the subset of 99 HIV-infected infants <4.5 months old followed longitudinally, hospital-diagnosed infants did not differ from PMTCT-diagnosed infants in time to ART initiation; however, hospital-diagnosed infants were >3 times as likely to die (HR = 3.1, 95% CI = 1.3-7.6).

**Conclusions:**

Among HIV-exposed infants, hospital-based testing was more likely to detect an HIV-infected infant than PMTCT testing. Because young symptomatic infants diagnosed with HIV during hospitalization have very high mortality, every effort should be made to diagnose HIV infections before symptom onset. Systems to expedite turnaround time at PMTCT EID sites and to routinize inpatient pediatric HIV testing are necessary to improve pediatric HIV outcomes.

## Background

Early infant HIV diagnosis and initiation of antiretroviral treatment (ART) are critical for infant survival [1,2]. A randomized trial in South Africa found that initiation of ART within the first 3 months of life, prior to disease progression, markedly improved infant survival compared to delayed ART [3]. However, early infant diagnosis (EID) and prompt initiation of ART remain uncommon [4-6]. The WHO estimates that in 2010 only 28% of HIV-exposed infants worldwide received a virologic HIV test within the recommended first 2 months of life [7]; fewer still returned for test results and initiated ART [4,5]. Thus, late identification of HIV-infected infants, who often present for care in a hospital setting when symptomatic, remains common, particularly in sub-Saharan Africa, which is home to >85% of the world’s HIV-infected children [8,9].

As PMTCT program effectiveness increases for women with access, fewer new infant infections will occur and be diagnosed through PMTCT-based EID systems [10,11]. Residual infant HIV infections will occur among women who do not attend PMTCT, those with poor attendance or adherence to PMTCT, or those who were HIV-uninfected at first antenatal visit and who subsequently acquired HIV during pregnancy or postpartum. Identifying alternative high yield testing sites to diagnose infant HIV infections can inform policy priorities for pediatric HIV testing. Efficient, appropriate targeted testing has the potential to detect and link infected infants to care. In this report, we compare HIV prevalence, test turnaround time, and outcomes of children receiving HIV testing in PMTCT programs versus in a tertiary care hospital in Nairobi, Kenya.

## Methods

### Ethics statement

The study was approved by the University of Washington Institutional Review Board (IRB) and the Kenyatta National Hospital (KNH)/University of Nairobi (UoN) Ethics and Research Committee (ERC). Written informed consent was obtained from each child’s primary caregiver.

### Study design and HIV testing

This analysis is based on recruitment, enrollment, and pre-randomization data from the Optimizing Pediatric HAART (OPH03) randomized trial (NCT00428116). Recruitment was conducted between 2007 and 2009 and was based at hospital pediatric wards at KNH and selected PMTCT sites at Nairobi City Council Clinics (NCCC). In PMTCT clinics, HIV-exposed infants <12 months of age were tested at 6 weeks of age and at any other time when they presented to the clinic after 6 weeks. In hospital pediatric wards, all infants who presented for care were tested for HIV. In hospital, infants were first tested by rapid HIV serologic test to determine whether they were HIV-exposed. Infants testing positive by rapid test in hospital or known to be HIV-exposed in PMTCT were tested by PCR to determine infection status. Infants who tested positive by PCR in hospital or PMTCT settings were referred to the study staff for enrollment, at which time their HIV status was confirmed by a second PCR test. PCR tests were conducted at the National Laboratory at the Centers for Disease Control and Prevention (CDC) in Nairobi.

### Subset followed longitudinally in OPH03 study

Infants aged less than 4.5 months were eligible for the OPH03 trial if they were HIV DNA positive and their caregiver planned to remain in Nairobi for at least 3 years and was willing and able to provide sufficient locator information [12]. Infants were ineligible if they had previous ART (except for PMTCT). Before 2009, infants suspected to have active tuberculosis were excluded. At enrollment, physical examination was performed, sociodemographic information and medical history were obtained, and blood samples were collected from infants. Viral loads were obtained by GenProbe assay [13], and CD4 count and CD4 percent were determined by flow cytometry. Caregivers received infant ART adherence counseling and infants initiated ART with lamivudine, zidovudine, and nevirapine, or ritonavir-boosted lopinavir if the infant had been exposed to nevirapine as part of PMTCT. Infants had monthly follow-up visits. Infant mortality data was collected from caregivers during these visits. Pre-randomization OPH03 study data, including data from enrollment to 2 years following ART initiation, were analyzed.

### Statistical analysis

Among infants in the recruitment screening cohort, prevalence of HIV was compared between hospital and PMTCT sites using a Chi-squared test, and the number needed to test (NNT) was calculated as inverse of HIV prevalence measures. Wilcoxon rank-sum tests were used to compare infant ages at HIV testing between hospital and PMTCT sites as well as turnaround time of HIV tests among HIV-infected children between hospital and PMTCT sites. In the longitudinally-followed subset of children enrolled in the OPH03 trial, time to death (overall, pre-, and post-ART initiation) and time to ART initiation were compared between diagnosis sites using log-rank tests and Cox proportional hazards regression. For overall and pre-ART analyses, time was days since enrollment; for post-ART analyses, time was days since initiation of ART. In mortality analyses, infants were censored at the time they were lost or randomized into the parent clinical trial. In the time to ART initiation analysis, infants were censored at the time they were lost, deceased, or randomized into the parent clinical trial.

All analyses were conducted using Stata 11.2 IC (StataCorp, College Station, TX). All tests were two-tailed with alpha = 0.05.

## Results

### HIV prevalence during recruitment screening

**Figure 1:**
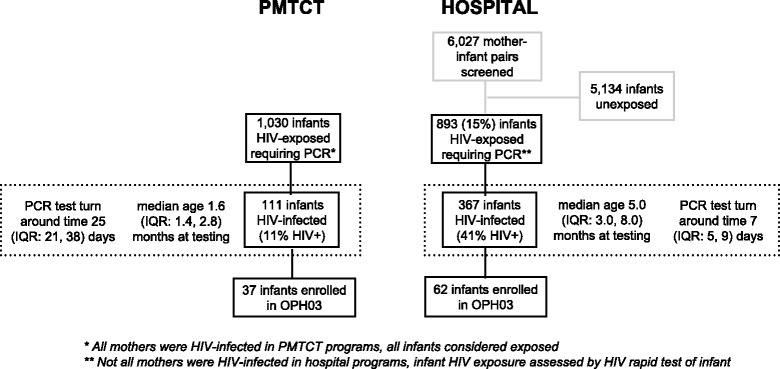
**Testing yield, infant ages, and turnaround time for infant HIV testing at hospital and PMTCT.** Infants were tested for HIV in PMTCT clinics and pediatric hospital wards as part of the parent clinical trial recruitment procedures. This figure shows the testing steps, test turnaround time, and infant age at testing among the two recruitment clinic types. Among HIV-exposed infants, HIV infection was more prevalent in hospital wards than in PMTCT clinics, infant age at testing was higher in hospital wards than in PMTCT clinics, and test turnaround time was shorter in hospital wards than in PMTCT clinics.

Among 7,057 mother-infant pairs with test results available from recruitment, 6,027 were screened in hospital pediatric wards and 1,030 were screened in PMTCT clinics; a total of 1,923 infants were HIV-exposed. In PMTCT, 111 (11%) of 1,030 HIV-exposed infants were HIV-infected. To identify one HIV-infected child at a PMTCT clinic, 9.1 HIV-exposed children would need to be tested using standard infant testing algorithms. Among all 6,027 children screened in hospital the prevalence of infant HIV infection was 6%, while among the 893 HIV-exposed infants, HIV prevalence was significantly higher than in PMTCT clinics at 41% (p < 0.001) (Figure 1). For each HIV-infected infant identified in hospital, 2.4 HIV-exposed infants or 16.4 infants of unknown HIV exposure status would need to be tested.

Among HIV-infected infants, those diagnosed in the hospital were significantly older than infants diagnosed in PMTCT clinics [median = 5 (IQR = 3, 8) versus 1.6 (IQR = 1.4, 2.8) months, respectively, p < 0.001]. However, time between testing and delivering infant test results to the caregiver was shorter among hospital-diagnosed infants than PMTCT-diagnosed infants [median = 7 (IQR = 5, 9) versus 25 (IQR = 21, 38) days, respectively, p < 0.001].

### Subset of longitudinally followed infants

Among the subset of 99 infants enrolled in the longitudinal cohort, 37 were diagnosed at PMTCT and 62 were diagnosed in a hospital setting (Figure 1). A majority of primary caregivers were the infants’ biological mothers (97%), most were in a monogamous marriage (78%), and were either unemployed and/or worked as housewives (83%). Most fathers were of unknown HIV status (as reported by the primary caregiver) (62%), and only 11% were reported to be HIV negative. Among infants with siblings, most had siblings of unknown HIV status (71%). At diagnosis, infants were, on average, just under 4 months old (median 3.7 months), had low CD4% (median 18%), high viral loads (median 6.5 log_10_ copies/ml HIV RNA) and nearly half (49%) were WHO clinical stage 3 or 4.

### Outcomes associated with infant diagnosis site

**Figure 2:**
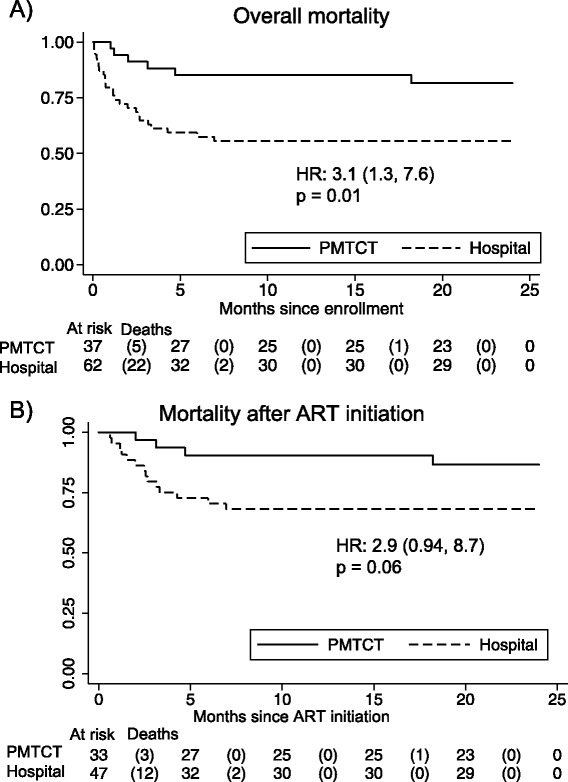
**Comparison of survival overall and after ART initiation of HIV-infected infants, by place of diagnosis (hospital vs. Prevention of Mother-to-Child Transmission [PMTCT] site): Kaplan-Meier Survival Analysis.** Infants enrolled in the parent clinical trial were followed prospectively; their mortality is compared in this graphic using Kaplan-Meier curves. Panel **A**: Overall mortality was significantly higher among the hospital-diagnosed infants than the PMTCT-diagnosed infants. Panel **B**: Differences in mortality persisted after ART initiation, with a trend towards significance.

Overall mortality rates and correlates of mortality have been previously reported for the full cohort [12]. Infants diagnosed in hospital were three times as likely to die as infants diagnosed through PMTCT (HR = 3.1, 95%CI = 1.3-7.6; Figure 2 panel A and Table 1) despite being similar ages due to longitudinal cohort age restrictions (p = 0.09). When adjusting for infant CD4% at enrollment, diagnosis in hospital remained a risk factor for death (aHR = 2.7, 95%CI = 1.1-6.8). Adjustment for WHO clinical stage was not appropriate given a high degree of correlation with infant diagnosis site; 75% of infants diagnosed in the hospital were classified as WHO Clinical Stage III or IV, versus just 8% of those diagnosed in PMTCT sites (p < 0.001). Following ART initiation, hospital-diagnosed infants had a trend for persistently higher mortality (HR = 2.9, 95% CI = 0.94-8.7; Figure 2 panel B and Table 1). Infants in the two groups did not differ in time to initiation of ART (p = 0.3).

**Table 1:** **Differences in Mortality and time to ART initiation between Hospital and PMTCT**

	**PMTCT**		**Hospital**			
**Outcome of Interest**	**N**	**Median (IQR) or n (%)**	**N**	**Median (IQR) or n (%)**	**HR (95%CI)**	**p-value**
Overall Mortality	37	6 (16%)	62	24 (39%)	3.1 (1.3, 7.6)	0.01
Mortality pre-ART	37	2 (5%)	62	10 (16%)	2.9 (0.64, 13)	0.2
Mortality post-ART	33*	4 (12%)	47*	14 (30%)	2.9 (0.94, 8.7)	0.06
Time to ART initiation (enrollment to ART initiation)	37		62		0.80 (0.51, 1.3)	0.3
Median days to ART initiation	33*	8 (7-20)**	47*	14 (7-20)**		

*Among 37 infants diagnosed in PMTCT, 2 died and 2 were lost to follow-up prior to initiating ART; among 62 infants diagnosed in hospital, 10 died, 1 was lost to follow-up, and 4 were withdrawn prior to initiating ART.

**Median time to initiation of ART among those children who initiated ART.

## Discussion

In this comparison of PMTCT and hospital HIV testing sites in Nairobi, Kenya, HIV-exposed infants tested in hospital were ~4 times as likely to be HIV-infected as those tested in the PMTCT clinic. Hospital-diagnosed infants were on average 3.5 months older than PMTCT-diagnosed infants at the time of testing and caregivers of HIV-infected hospital-diagnosed infants received infant HIV test results almost 3 weeks faster than PMTCT-diagnosed infants. Hospital-diagnosed infants in the longitudinal cohort had 3-fold higher mortality compared to PMTCT-diagnosed infants, likely due to differences in the proportion presenting with WHO clinical stage III/IV infections. Together, these findings underscore the need for better strategies for prompt pediatric HIV detection and treatment.

Among HIV-exposed infants in this study, there was a significantly higher prevalence of infant HIV in the hospital setting than in PMTCT programs. Our findings are consistent with recent reports noting that HIV testing in hospital pediatric wards identifies a large number of HIV-infected children in high prevalence areas [14,15]. As PMTCT programs are increasingly successful, the majority of infants diagnosed with HIV will be from mothers who do not access PMTCT services [10,11]. However, in contrast to routine PMTCT EID programs, in-patient pediatric HIV testing is not systematically implemented due to inadequate staffing, test shortages, and unavailability of rapid infant HIV DNA assays [16]. Models of task-shifting with peer mentors may facilitate in-patient pediatric HIV testing [15,17].

The extremely rapid progression of infant HIV means that a few months delay in detection of HIV is associated with significantly increased mortality. In our study, turnaround times for PCR results were significantly longer in PMTCT clinics than in hospital, consistent with other studies noting long turnaround times for PCRs in routine PMTCT clinics [18-20]. Some studies have noted mortality among infants before test results are available [20]; long turnaround times present an opportunity for improving PMTCT systems to more quickly deliver test results and link infected children to care. Novel best practices to decrease the delivery time of EID results to providers and parents are potentially promising and range from mobile SMS and telephone technology use to improved referral systems [21,22]. These will be critical to facilitate prompt diagnosis and treatment of HIV-infected infants, which in turn will prevent hospitalization and mortality.

We found that infants diagnosed in hospital were older than their PMTCT-diagnosed counterparts, which is consistent with previous studies comparing inpatient to outpatient settings [23]. Attrition of children at risk for HIV infection from PMTCT programs and subsequent presentation in hospital has been noted in other settings [24]. In addition, some children may acquire HIV via breastfeeding after an initial negative HIV test at 6 weeks of age. Infants who were diagnosed in hospital were more than 3 times as likely to die as their PMTCT-diagnosed counterparts. Mortality risk among hospital-diagnosed infants persisted, albeit not significantly, after infants initiated ART, consistent with previous reports noting that infants who are already symptomatic at ART initiation do not respond as effectively to ART as their asymptomatic counterparts [12,25] and reports noting higher mortality among children identified in inpatient versus outpatient settings [26].

Strengths of our study include longitudinal and systematic ascertainment of infant mortality following diagnosis, high retention, and detailed ascertainment of caregiver characteristics. However, the study had several limitations. The data available on infants screened but not enrolled was limited and our enrolled cohort was small, potentially limiting our ability to detect differences in outcomes such as time to ART initiation and post-ART mortality. As participants in a randomized trial, the enrolled mother-infant pairs are likely to have limited generalizability. Finally, PMTCT coverage and EID implementation systems are changing rapidly and these historic data may not reflect current prevalence of HIV in PMTCT programs and hospital.

## Conclusions

There are many drop-off points along the EID “cascade” from timing of first testing to receipt of results and prompt ART initiation [4,5,23]. Our study reinforces the importance of diagnosing infants early before they become symptomatic and their survival even with ART is compromised. Lapses in the infant EID system, delays in conveying positive infant results and in timely initiation of ART, and lack of infant testing among women who did not receive antenatal HIV testing, or who subsequently acquired HIV in pregnancy or postpartum, or who did not access MCH or PMTCT programs all lead to preventable infant mortality. Ultimately, a rapid point of care assay would be the most useful innovation to ensure timely detection and treatment of infants.
